# Ecological dynamics of influenza A viruses: cross-species transmission and global migration

**DOI:** 10.1038/srep36839

**Published:** 2016-11-09

**Authors:** Hongguang Ren, Yuan Jin, Mingda Hu, Jing Zhou, Ting Song, Zhisong Huang, Beiping Li, Kaiwu Li, Wei Zhou, Hongmei Dai, Weifeng Shi, Junjie Yue, Long Liang

**Affiliations:** 1 State Key Laboratory of Pathogen and Biosecurity, Beijing Institute of Biotechnology, Beijing 100071, China; 2 Institute of Pathogen Biology, Taishan Medical College, Taian 271000, China

## Abstract

A comprehensive study of cross-species transmission and inter-regional migration would provide insights into the global ecology of influenza A viruses (IAVs). To this end, we assembled 17,241 non-redundant IAV whole-genome sequences with complete epidemiological information. We hierarchically divided the movements of IAVs into the cross-species transmission in each region and the inter-regional migration driven by each host species. We then systematically identified the potential cross-species transmission and inter-regional migration events. Cross-species transmission networks were obtained for each gene segment of the IAVs. Waterfowl, domestic birds and swine showed higher degrees of connection than did other species in all of the transmission networks. East Asia and Southeast Asia were hot regions for avian-mammal transmissions. Swine and migratory birds were the dominant species for global virus delivery. The importance of swine was reemphasized because it has not only provided an environment for adaptive evolution during the avian-human transmission of IAVs (as incubators) but also served as a key species for the global dissemination of the viruses (as carriers). Therefore, monitoring the global live trade of swine and survey of migratory birds along flyways would be beneficial for the prevention and control of IAVs.

Both the annual epidemics and occasional pandemics caused by Influenza A viruses (IAVs) pose a serious threat to public health. IAVs are among the most common causes of human respiratory infections and lead to high morbidity and mortality^1^. They have been isolated from many hosts, including human, pigs, horses, dogs, and both wild and domestic birds. Phylogenetic studies have revealed that the viral genes form aquatic birds are often thought to be reservoirs for all IAVs from other species^2^. The spillover of avian influenza A viruses into humans often leads to pandemics with high mortality^3^,^4^,^5^. The genetic barriers between host species limit the free transmission of IAVs from avian species to mammals^6^. To date, certain subtypes have become established in mammals, such as the H1N1 (including seasonal H1N1 and H1N1pdm09) and H3N2 viruses in humans. In addition, there have been at least five HA subtypes (H5, H6, H7, H9 and H10) of avian influenza A viruses that are able to infect humans^4^,^7^,^8^,^9^,^10^,^11^,^12^.

In general, the ecological scope of IAVs consists of the spatial distribution and the infected host range. Globally, IAVs can be carried anywhere by means of the hosts’ global movements, including the global air travel of human beings, the migration of wild birds, and the intercontinental live swine trade. The migration patterns of some subtypes of IAVs, especially the influenza A/H3N2 viruses and avian influenza A/H5N1 viruses, have been well characterized^9^,^13^,^14^,^15^,^16^,^17^,^18^. Locally, IAVs may infect new hosts and become established through adaptive evolution. Previous studies of cross-species IAVs were mainly focused on specific amino acid mutations in the haemagglutinin (HA) protein, which might lead to potential changes in receptor-binding preference, and consequently altered host specificity and tropism^19^. However, the ecological dynamics of all IAVs, including cross-species transmission and global migration, remain poorly understood.

To characterize the cross-species nature and the migration of IAVs on a global scale, we conducted a comprehensive analysis of 17,241 well-documented full-length IAV genomes. Cross-species transmission networks of IAVs were then estimated from the dataset, providing a schematic diagram of the transmission activities of IAVs among species. We hierarchically analyzed the possible cross-species transmission events for each region and the migration events driven by each species, which allowed us to identify the regions that were enriched with cross-species transmissions and the key host species that are responsible for the global migration of IAVs. Our findings provide insight into the prevention and control of the worldwide spread of IAVs.

## Results

### Summary of IAV Genomes

A total of 17,241 (accounting for all full-length IAV sequences available from GenBank as of January 30, 2015) non-redundant IAV whole-genome sequences with complete epidemiological information were assembled, such as the isolation date, host and location of sampling (Supplementary Data 1). To our knowledge, these are the largest geographically and temporally informative IAV datasets assembled to date.

**Figure 1:**
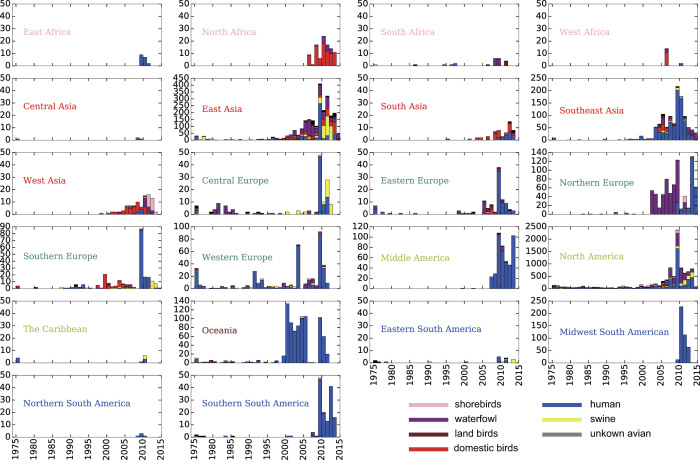
Statistics of Global IAV genome. Genome numbers sampled each year in each region of the world are shown. Species of the viruses are indicated by different colors.

The sampling locations were divided into 22 groups at the sub-continent level according to the administrative division (Fig. 1, Supplementary Table 1). Although IAVs have been detected in hosts from more than 100 species, >99 percent of these genome sequences are sampled from avian hosts, swine and humans (Supplementary Data 1). For avian hosts, more than 100 species of wild birds have been found to harbour IAVs^20^. According to their living habits, we further grouped the avian hosts into four sub-classes: shorebirds, waterfowl, land birds and domestic birds (Methods, Supplementary Table 2). The term ‘species’ used in this paper refers to one of the six host classifications, including human, swine and the four sub-classes of avian hosts.

The sampling frequency reached the highest in the year 2009 for almost all regions (Fig. 1), when the H1N1pdm09 viruses emerged. Surveillance of IAVs in East Asia and North America was more frequent and covered more hosts than the other regions. On the one hand, the sampling biases of the data set might have been related to the different surveillance intensities in different regions. On the other hand, these biases might actually reflect the different activity levels of IAVs.

### Construction of the cross-species transmission networks of IAVs

**Figure 2:**
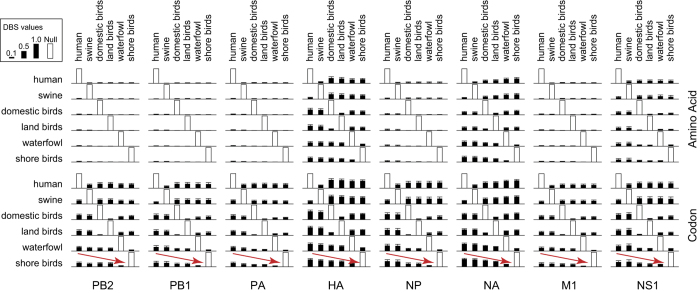
Mean DBS values of IAVs at the amino acid and codon levels. Filled bars show the mean defined distances between each pair of species of each gene segment of the IAVs at the amino acid and codon levels, respectively. Standard deviations were plotted on top of each bar. A possible directional trend between species was depicted as arrows in the figure.

Generally, IAVs sampled from different species exhibited different molecular characteristics. To assess the differences of IAVs among species, we defined the distance between species (DBS, see Methods) as the average difference of amino acid (or codon) distribution in each loci between viruses sampled from different species. Regardless of the virus subtype, we systematically calculated the mean distance between viruses sampled from each pair of different species. (Fig. 2, Supplementary Figures). The DBS values for pairs of species were not observable for the internal genes at the amino acid level, except for the HA and NA segments. However, the DBS values were much higher at the codon level, which implied species-specific molecular evolution of IAVs at the nucleotide level. Furthermore, the DBSs were remarkably different for the same gene segment. For example, the value between human and shorebirds were larger than that between human and swine. These DBS values highlighted the need to analyze the cross-species transmission patterns of IAVs.

**Figure 3:**
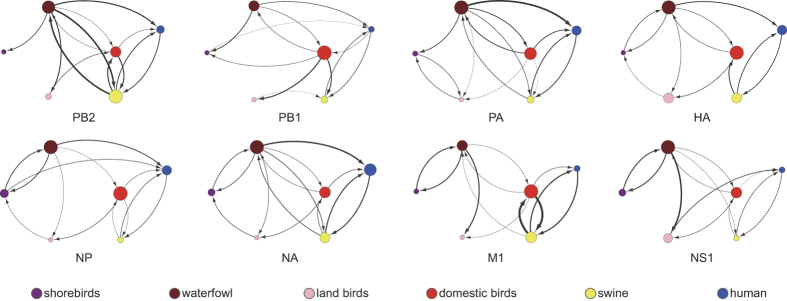
Cross-species transmission networks of IAVs. The transmission linkage of species constructed using BEAST for each gene segment of IAVs. Each colored node represents a specific species. Lines and arrows represent transmissions and directions with Bayes factor >3. Thickness of lines represents the relative transmission rate between two species. The size of each node is proportional to the sum of the relative transmission rates of the species.

To explore the transmission patterns between species, we performed a species-associated phylogenetic analysis for each gene segment using BEAST^21^. The species of the sequences was used as a trait to infer the transmission network of IAVs. We sub-sampled the sequences (the sub-sampling method can be seen in the Methods) for each gene segment to reduce the computational load (the xml file for BEAST can be found in Supplementary Data 2). Confidential transmission links (with Bayes factors >3) between species were abstracted to construct the cross-species transmission network for each gene segment (Fig. 3). It should be noted that the aim was not to determine the origins of IAVs; instead, we sought to construct the confidential patterns that appeared in the cross-species transmission history of IAVs. Different gene segments showed different topologies and connecting strengths in the network, but several common transmission patterns could be obtained. First, transmissions between human and swine, and between waterfowl and domestic birds were estimated to be bidirectional for nearly all eight gene segments, indicating that frequent transmission events might have occurred between these species. Second, waterfowl, domestic birds and swine acted as key hub species, indicated by the higher degree of connectivity in the transmission networks. Third, although the networks were highly connected, several species pairs were infrequently found to be related, including shorebirds and domestic birds and land birds and swine, which might have been due to the isolation of living environments for these species.

### Hot regions for cross-species transmission of IAVs

**Figure 4:**
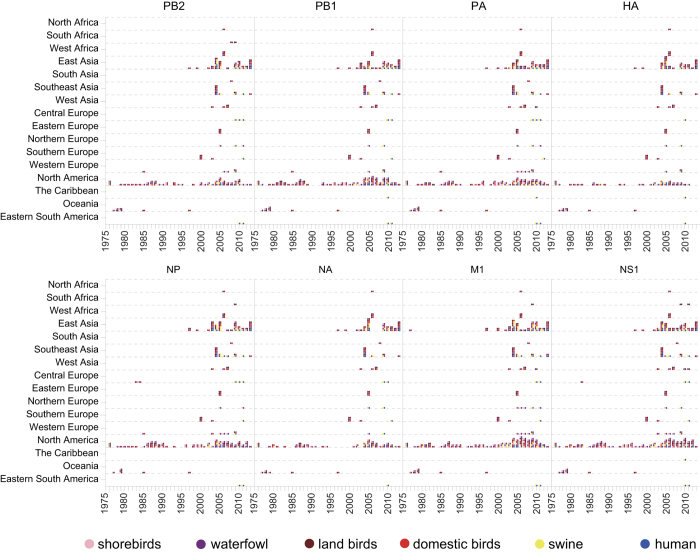
Local cross-species transmission of IAVs in each region. Circles with colors represent different species, respectively. A possible transmission event in the same region is indicated by a pair of circles placed vertically in the same year. Multiple cross-species transmission in the same region in the same year are piled up vertically. Regions without identified transmission events are hided.

To further identify the possible transmission events of IAVs among different hosts in different regions, we hypothesized that the transmissions of IAVs were hierarchical and consisted of regional cross-species transmission and inter-regional migration. Taking the cross-species transmission phenomenon as the co-appearance of highly similar (>99 percent similarity in this paper) sequences in different species in a specific region within a short time window (<3 years in this paper) (Methods), we summarized the possible transmission patterns for each region on the basis of the whole data set without resampling (Fig. 4, Supplementary Table 3).

All eight gene segments showed similar species transmission patterns. More cross-species transmission events of IAVs were detected in North America, East Asia and Southeast Asia compared to other regions. Since the beginning of this century, the host jump activities of IAVs in these regions have increased. Furthermore, there were more avian-mammalian transmissions in Asia, whereas other regions were enriched by intra-avian and intra-mammalian transmissions. East Asia and Southeast Asia were estimated to be hot regions for avian-mammal transmission of IAVs over the last decade, which coincided with their role as the epicenters for recent IAV outbreaks^5^,^12^,^22^. Domestic birds, especially those traded in live poultry markets in these areas, have been criticized as the source of IAVs that could infect humans^23^,^24^. The H9N2 viruses have been reported to serve as a progenitor for novel human avian influenza viruses^23^,^24^,^25^,^26^, and the corresponding poultry-human transmission has also been captured in our results (Fig. 4, Supplementary Table 3). The early cross-species transmission from avian hosts to swine^27^ was also identified in our results.

### Key species for the global migration of IAVs

**Figure 5:**
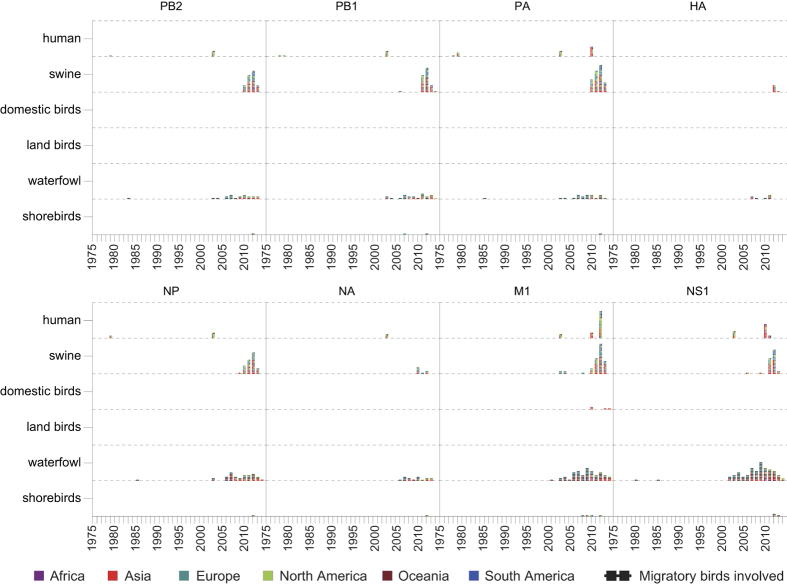
Inter-regional migration of IAVs driven by each species. Squares with colors represent different continents, respectively. Regions belonging to the same continent were depicted with the same color. A possible migration event for the same species is indicated by a pair of squares placed vertically in the same year. Multiple inter-regional migrations in the same species for the same year are piled up vertically. Migrations that have been partially contributed by migratory birds are indicated by a horizontal line crossing the two regions.

IAVs can be carried to any location to which the hosts travel. Here, we examined the possible migration events of IAVs driven by each host species. Similar to the definition of local cross-species transmission, a possible migration event was defined as the co-appearance of highly similar (>99 percent similarity in this paper) sequences in the same species in different regions within a short time window (<3 years in this paper). We then investigated the possible migration patterns of IAVs among the six host species (Fig. 5, Supplementary Table 4). In this colouring strategy, regions that belong to the same continent were depicted using the same colour.

Analysis of the six internal gene segments showed similar inter-regional migration patterns driven by the same host species (Fig. 5). Swine and waterfowl were shown to be the dominant species for the global dispersal of IAVs, as more migration events for these two species have been identified than other species. Specifically, IAVs carried by swine have been exchanged among Asia, Europe and North America. This result coincided with a recent study^28^ that the global live swine trade strongly predicted the spatial dissemination of swine influenza A viruses, with Europe and North America acting as sources of the viruses in Asian countries. We further investigated the migrations driven by avian hosts (mostly waterfowl and some shorebirds) and found that more than 85% (228/268) of these avian-driven migration patterns were associated with migratory birds (Fig. 5).

## Discussion

IAVs are expanding their host range, especially in mammals, which often poses threats to public health. Traditional cross-species transmission studies of IAVs often aimed to determine the specific amino acid polymorphisms in the HA protein of the viruses that determine the binding-specificities to different hosts. Regardless of the subtypes of IAVs, our comprehensive analysis of IAV genomes revealed both the transmission networks among species and the dominant factors affecting both the global and local ecology of IAVs.

In the cross-species transmission networks, the two bidirectional transmissions (human and swine, waterfowl and domestic birds) that were estimated to be conserved for all eight gene segments implied a relatively lower cross-species barrier for closely related species. Aquatic birds (including shorebirds and waterfowl) have been reported to be natural reservoirs of IAVs, which allowed domestic birds and swine to have central roles in the transmission of the viruses from avian to humans. The connections between domestic birds and swine seemed to be critical for IAV adaptation from avian to human. The polyculture mode of poultry and pigs in East Asia might have facilitated the adaptive evolution process. Because both species are major human diet sources, we highly recommend that the farming of poultry and pigs should be separated.

Species-specific movements across regions help viruses expand their spatial range. The discovery of swine as a dominant species in the global delivery of the viruses was in accordance with a recent study that described the role of the global live swine trade in the formation of the global ecology of swine influenza A viruses^28^. In addition, swine are often reported to be intermediate hosts and ‘mixing vessels’ by facilitating the reassortment of genes from different IAVs^29^. Thus, more attention should be paid to the inspection and quarantine of swine during international trade. Migratory birds have been reported to play important roles in the geographic spread of zoonotic agents^30^, especially avian influenza A viruses^31^,^32^. It has been previously demonstrated that the spread of HPAI H5N1 correlates with bird migration networks^9^,^33^. Our work also revealed that migratory birds (mainly waterfowl) have played an important role in globally disseminating the viruses. Surveillance of these migratory birds along flyways will be beneficial for the prevention and control of IAVs.

The active IAV cross-species transmission events, especially avian-mammal transmissions in East and Southeast Asia, have made these regions hot-spots for the leakage of avian influenza A viruses into humans. In North America, the cross-species transmission of IAVs mainly occurs between avian species. However, attention should be paid to the risk of avian influenza viruses potentially infecting mammals. After all, the most recent pandemic influenza viruses (H1N1pdm09) were genetically and antigenically similar to the earlier swine A(H1N1) viruses circulating in North America^34^.

It should be noted that sequence data combined with well-documented epidemiological information have made this analysis possible and will benefit future studies. Therefore, we suggest that researchers generating viral sequences should provide detailed sampling information when submitting the sequences to public databases. In particular, IAVs sampled from avian hosts should attach both a formal scientific name and common name together with the order and family information of the bird, instead of just using ‘Avian’.

To conclude, we have systematically studied the factors that may have affected the global ecology of IAVs, which will help guide the prevention and control of IAVs on a global scale.

## Methods

### Sequence handling and host classification

We retrieved full-length genome sequences (as of January 30, 2015) from the Influenza Virus Resources at the National Centre for Biotechnology Information (NCBI) ( http://www.ncbi.nlm.nih.gov/genomes/FLU/FLU.html). Sequences with illegal nucleotide acids in the codon regions and those with unclear sampling locations or hosts were excluded. Artificial sequences were also excluded. Altogether, we obtained 17,241 high-quality influenza viral genomes. Details for the sequences can be found in Supplementary Data 1. Multiple sequence alignment for each gene segment was performed using MAFFT v7.058^35^.

Bird names were abstracted from the strain name of the avian influenza A virus sequences. Based on their living habits, we further classified the birds into four groups (namely the species in this paper) guided by the taxonomic categories of the birds: domestic birds, land birds, waterfowl and shorebirds. Regarding the migratory information of the birds, we referenced the species list from the Convention on Migratory Species and the collection of Agreements and Memoranda of Understanding^36^. Detailed classification of avian influenza A virus genomes can be found in Supplementary Table 2.

### Distances between Species

In this paper, the distances between two species were defined as the sequence differences sampled from the two species. For each segment, we calculated the amino acid (and codon) distributions for each species in each loci. For example, the amino acid distributions of species *A* and species *B* at loci *i* in the segment *seg*_*X*_ were 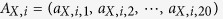 and 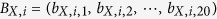, where *a*_*X*,*i*,*j*_ was the number of the *jth* amino acid at loci *i* in the sequences of species *A* divided by the total number of sequences of species *A*. Then, the distance between species *A* and species *B* at loci *i* in the segment *seg*_*X*_ was calculated as
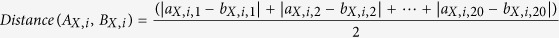


A similar methodology was used to calculate the codon based distances, except that the possible codon number is as high as 64 (instead of 20 for amino acids). The distance between each pair of species in each segment at each loci can be found in Supplementary Figs S1–S16. The mean distances for each loci in the same segment were shown in Fig. 2.

### Cross-species transmission network construction

We used Fasttree^37^ to generate an original tree of all the sequences for each gene segment. The subsampling of the sequences was performed by deleting the leaves that had shorter terminal branch lengths. We also attempted to delete the leaves with companies to represent their position in the trees without destroying the overall topology of the original trees. The subsampling process was repeated until a computable scale for BEAST was reached. The left sequences were then used to do the following Bayesian evolutionary analysis.

Just like the phylogeographic reconstruction method in BEAST using the locations of sequences to infer the migration networks, we used the species of the sequences to infer the potential transmission events among species for IAVs. A continuous time Markov Chain (CTMC) over discrete sampling species was employed. The sequences were grouped into 6 species (human, swine, domestic birds, land birds, waterfowl and shorebirds). Bayesian Markov chain Monte Carlo analysis was run for 200 million steps, sampled every 10,000 steps and the first 10% of which were removed as burn-in. Bayes factor tests were performed to provide statistical support for potential transmission routes between different species using SPREAD v1.0.6^38^. The Bayes factors for rates were derived from a Bayesian stochastic search variable selection procedure, and the phylogenetic linkage between species was constructed by routes with Bayes factor values >3.

### Identifying cross-species transmission and inter-regional migrations

In this paper, the co-appearance of two sequences with a >99% sequence similarity sampled from two different species in the same region in less than 3 years are defined as possible cross-species transmission. The timestamp of the transmission event was approximately estimated to be the later sampling time of the two sequences. For the same class of transmission events in the same region between *specie*_*i*_ with *subtype*_*i*_ and *specie*_*j*_ with *subtype*_*j*_, where *specie*_*i*_≠*specie*_*j*_, only the earliest one was recorded.

Similarly, the co-appearance of two sequences with a >99% sequence similarity sampled from the same species in two different regions in less than 3 years is defined as a possible inter-regional migration. The timestamp of the migration event is estimated to be the later sampling time of the two sequences. For the same class of migration events of the same species between *region*_*i*_ with *subtype*_*i*_ and *region*_*j*_ with *subtype*_*j*_, where *region*_*i*_≠*region*_*j*_, only the earliest one was recorded.

Detailed information of the identified regional cross-species transmission and inter-regional migration events can be found in Supplementary Tables 3 and 4.

## Additional Information

**How to cite this article**: Ren, H. *et al.* Ecological dynamics of influenza A viruses: cross-species transmission and global migration. *Sci. Rep.* **6**, 36839; doi: 10.1038/srep36839 (2016).

**Publisher’s note:** Springer Nature remains neutral with regard to jurisdictional claims in published maps and institutional affiliations.

## Supplementary Material

Supplementary Information

Supplementary File 1

Supplementary File 2

Supplementary File 3

Supplementary File 4

Supplementary File 5

Supplementary File 6

Supplementary File 7

Supplementary File 8

Supplementary File 9

Supplementary File 10

Supplementary File 11

Supplementary File 12

Supplementary File 13
