# Supplementary material for: Ecological dynamics of influenza A viruses: cross-species transmission and global migration
Source: Sci Rep. 2016 Nov 9;6:36839. doi: 10.1038/srep36839 (PMC5101809; doi:10.1038/srep36839)

Ecological dynamics of influenza A viruses: cross-species transmission and global migration

Hongguang Ren[1], Yuan jin[1], Mingda Hu[1], Jing Zhou[1], Ting Song[1], Zhisong Huang[1], Beiping Li[1], Kaiwu Li[1], Wei Zhou[1], Hongmei Dai[1], Weifeng Shi[2†], Junjie Yue[1†] & Long Liang[1†]

[1] State Key Laboratory of Pathogen and Biosecurity, Beijing Institute of Biotechnology, Beijing 100071, China.

[2]Institute of Pathogen Biology, Taishan Medical College, Taian 271000, China.

[†]Corresponding authors.

Correspondence and requests for materials should be addressed to W.S. (email: shiwf@ioz.ac.cn), J.Y. (email: yue_junjie@126.com), or L.L. (email: ll@bmi.ac.cn).

Supplementary information

Supplementary Figures

Supplementary Figures S1-S16. Detailed amino acid and codon distance between each pair of species on each loci for each segment of the IAVs

Distance values were indicated by colors: red bars represent that the value is large than 0.80; orange bars represent that the value is between 0.50 and 0.8; black bars represent the value is smaller than 0.50.

Supplementary Tables

Supplementary Table 1. Country classifications at sub-continent level

Supplementary Table 2. Genomes sampled from avian and detailed avian classifications

Supplementary Table 3. Detailed regional cross-species transmission events

Supplementary Table 4. Detailed inter-regional migrations driven by each species

Supplementary Data

Supplementary Data 1. Detailed genome Information

The information of each genome is included in the line started with symbol '>', and the ten segments of each genome were listed under the information line. Accession number of the corresponding protein and coding region for each segment were also listed.

Supplementary Data 2. Xml files for BEAST

Supplementary Figure S1

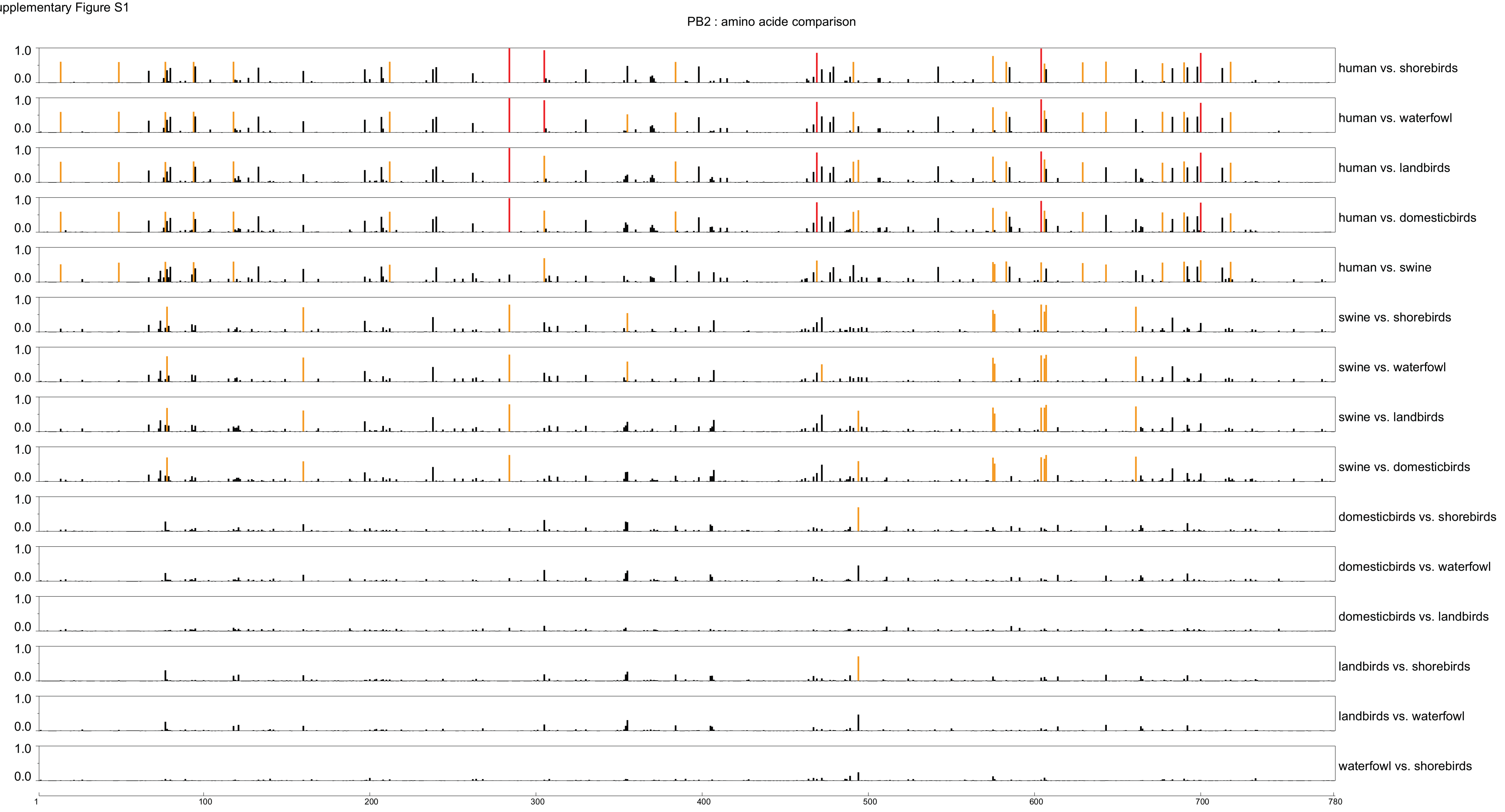

Supplementary Figure S2

PB2 : codon comparison

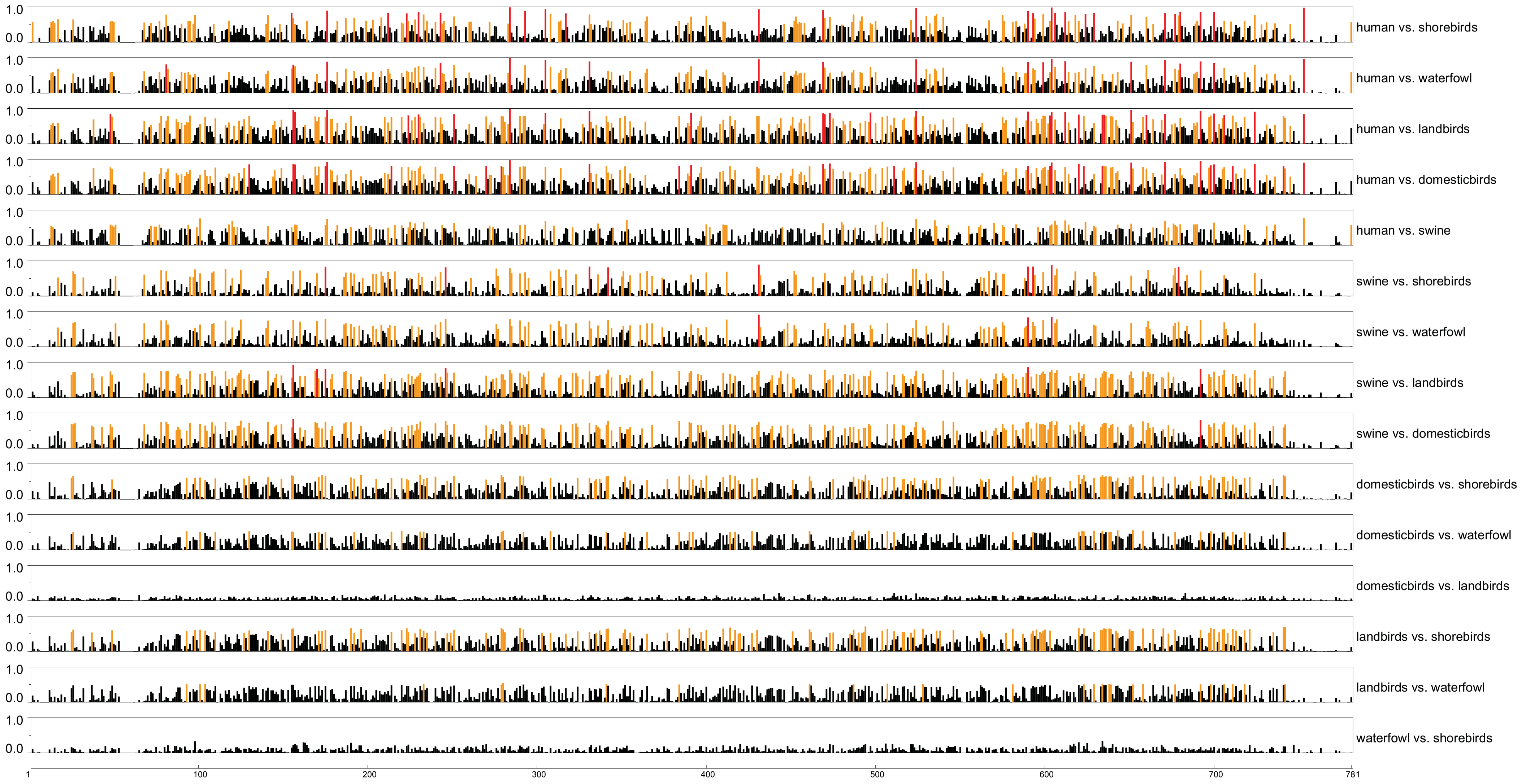

Supplementary Figure S3

PB1 : amino acide comparison

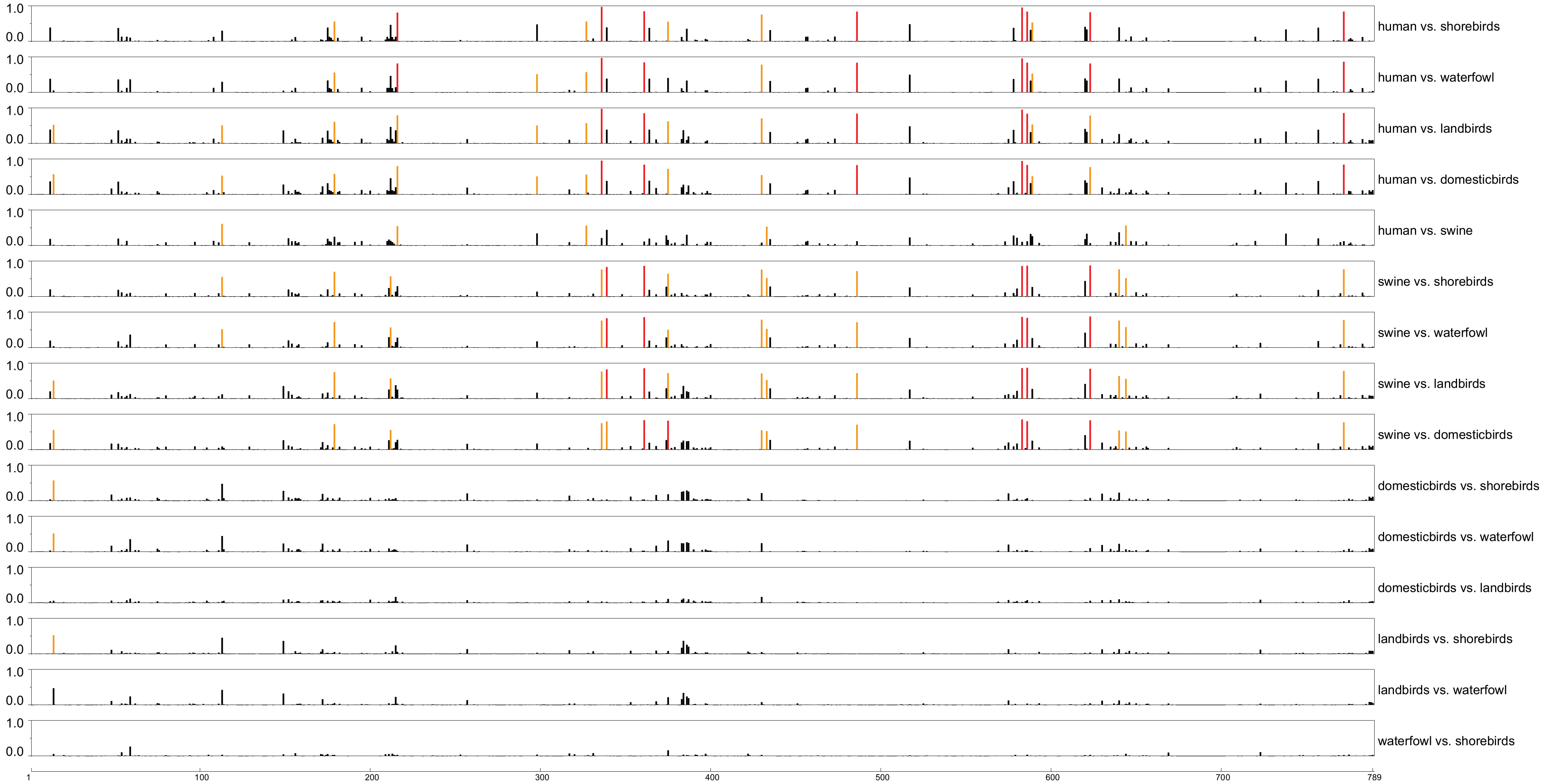

Supplementary Figure S4

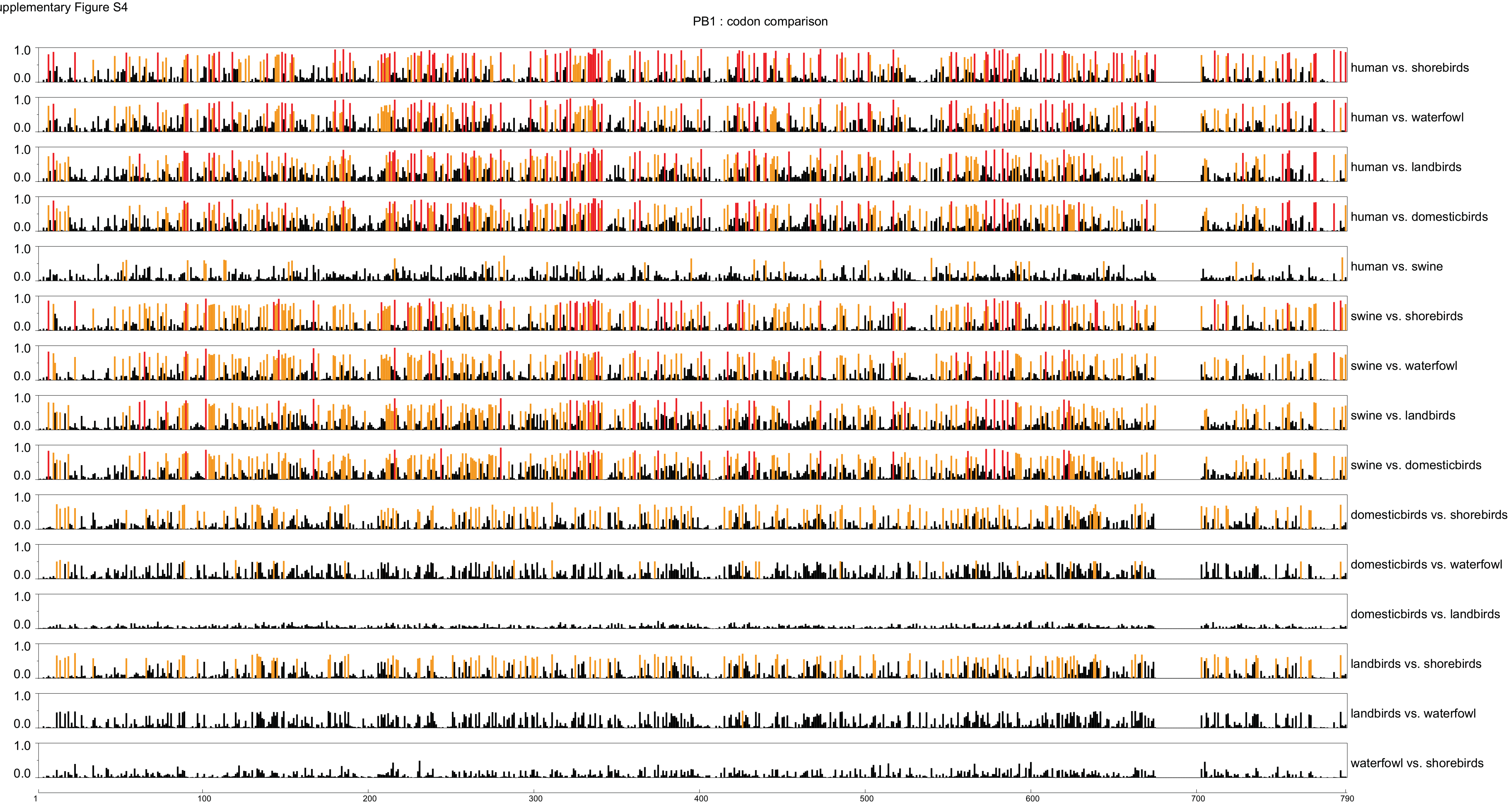

Supplementary Figure S5

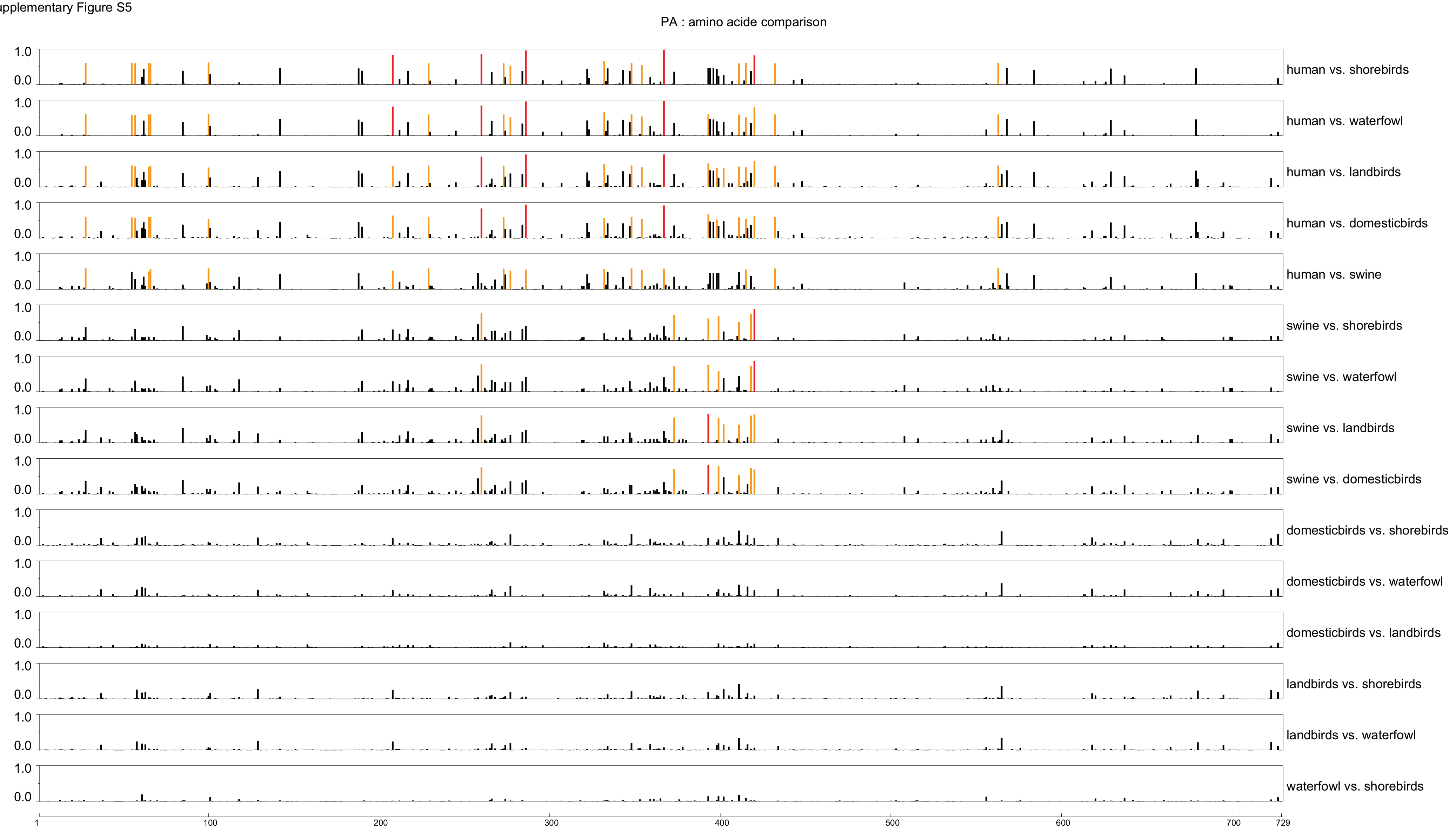

Supplemntary Figure S6

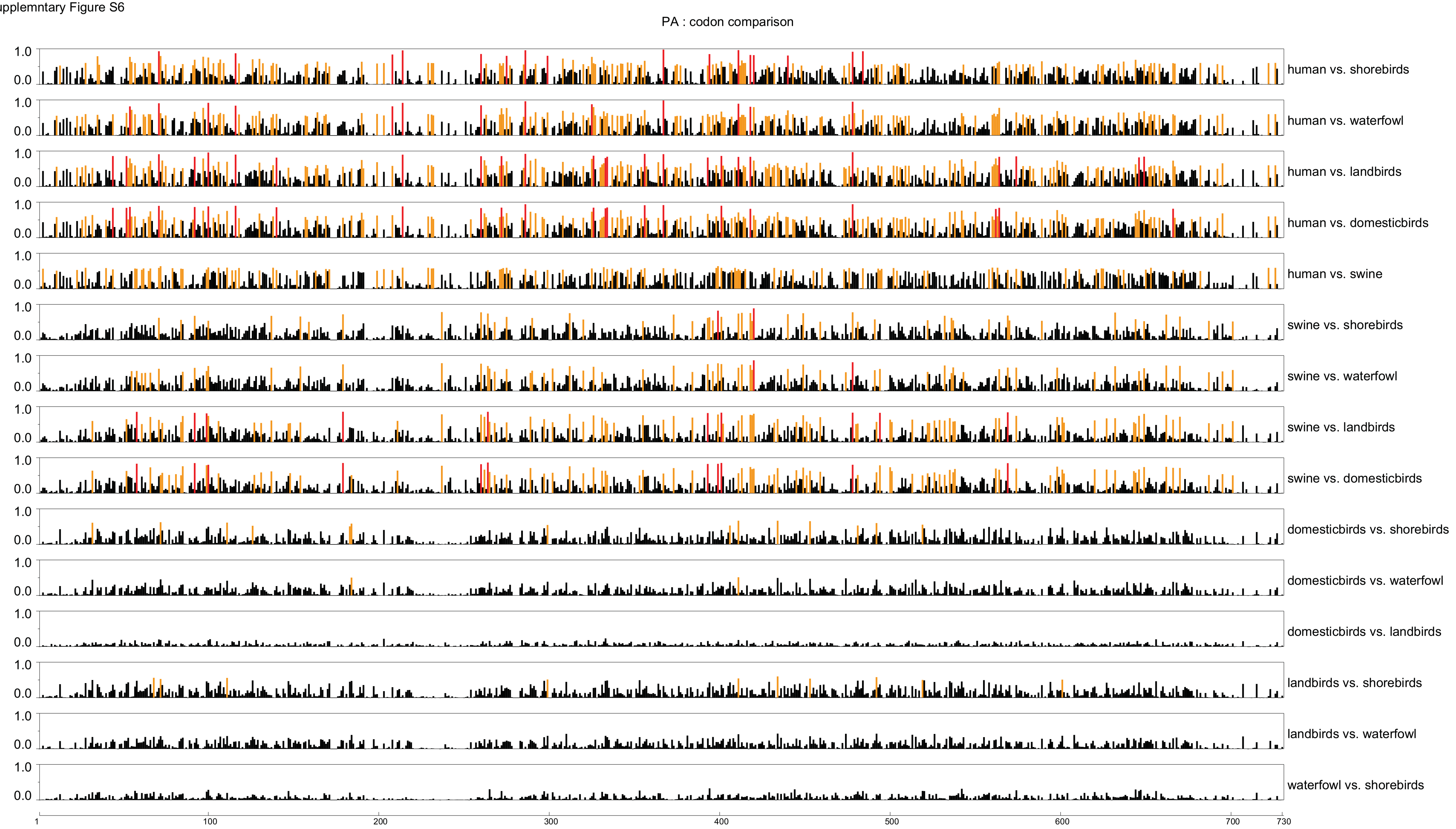

Supplementary Figure S7

HA : amino acide comparison

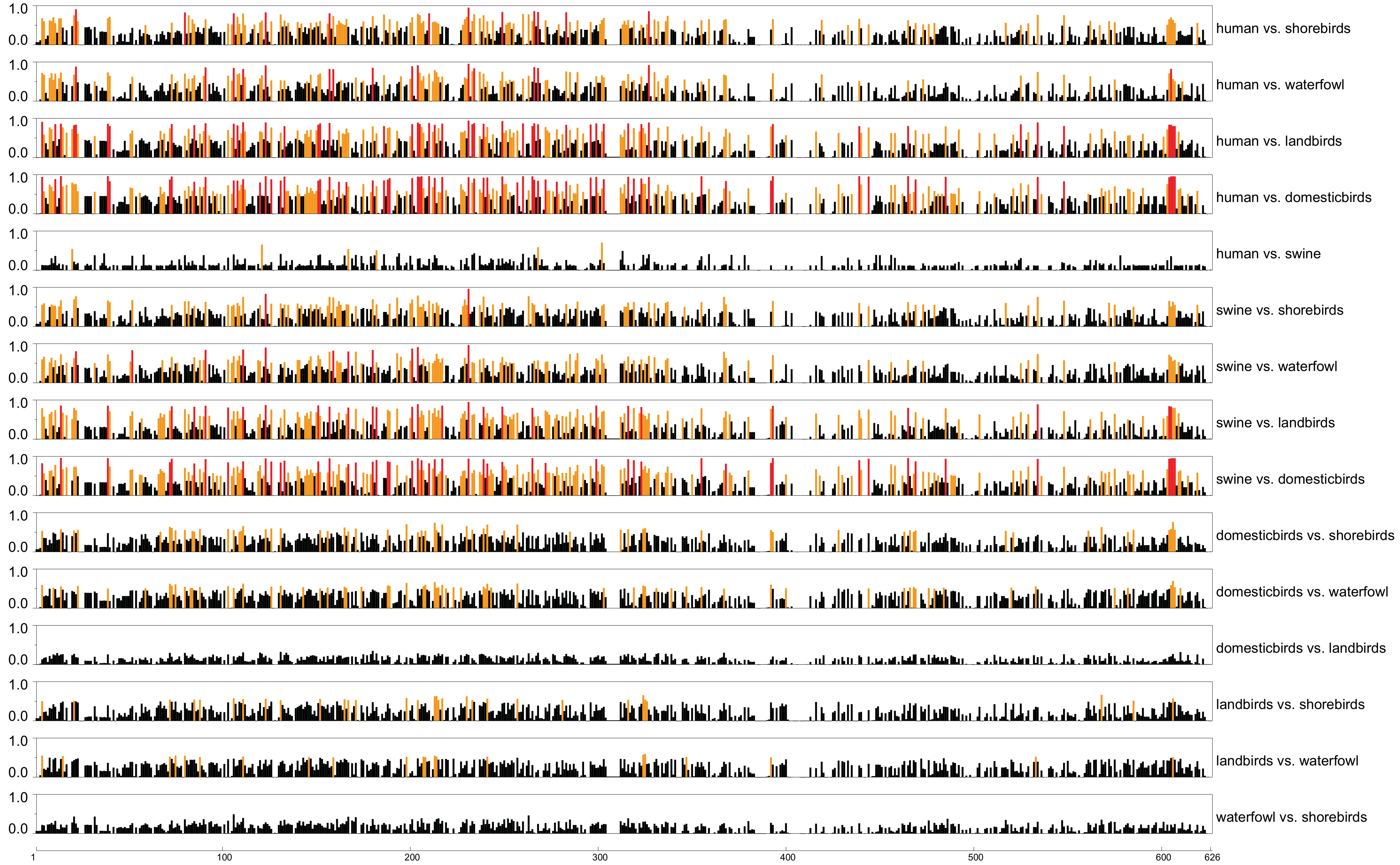

Supplementay Figure S8

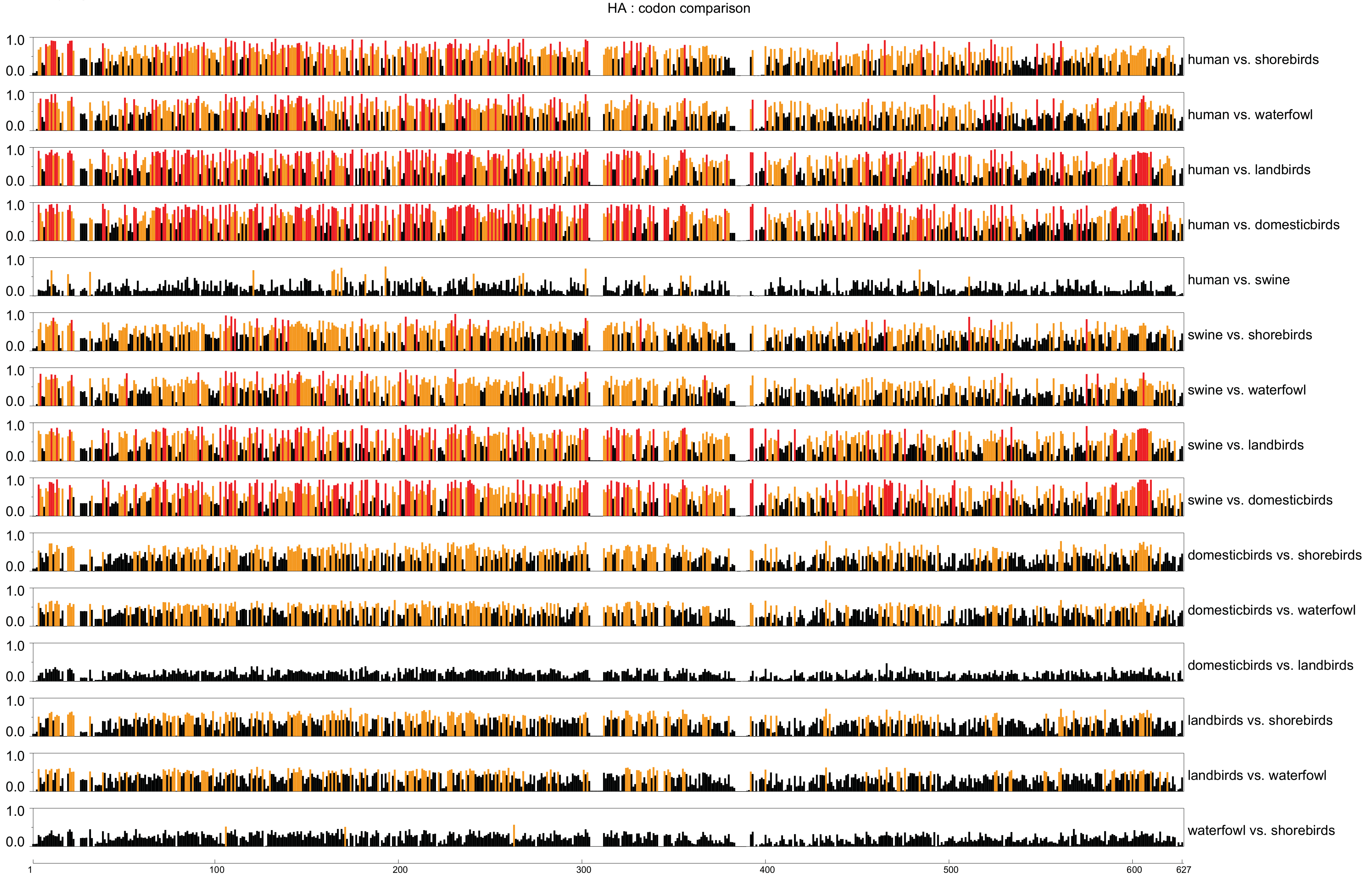

Supplementary Figure S9

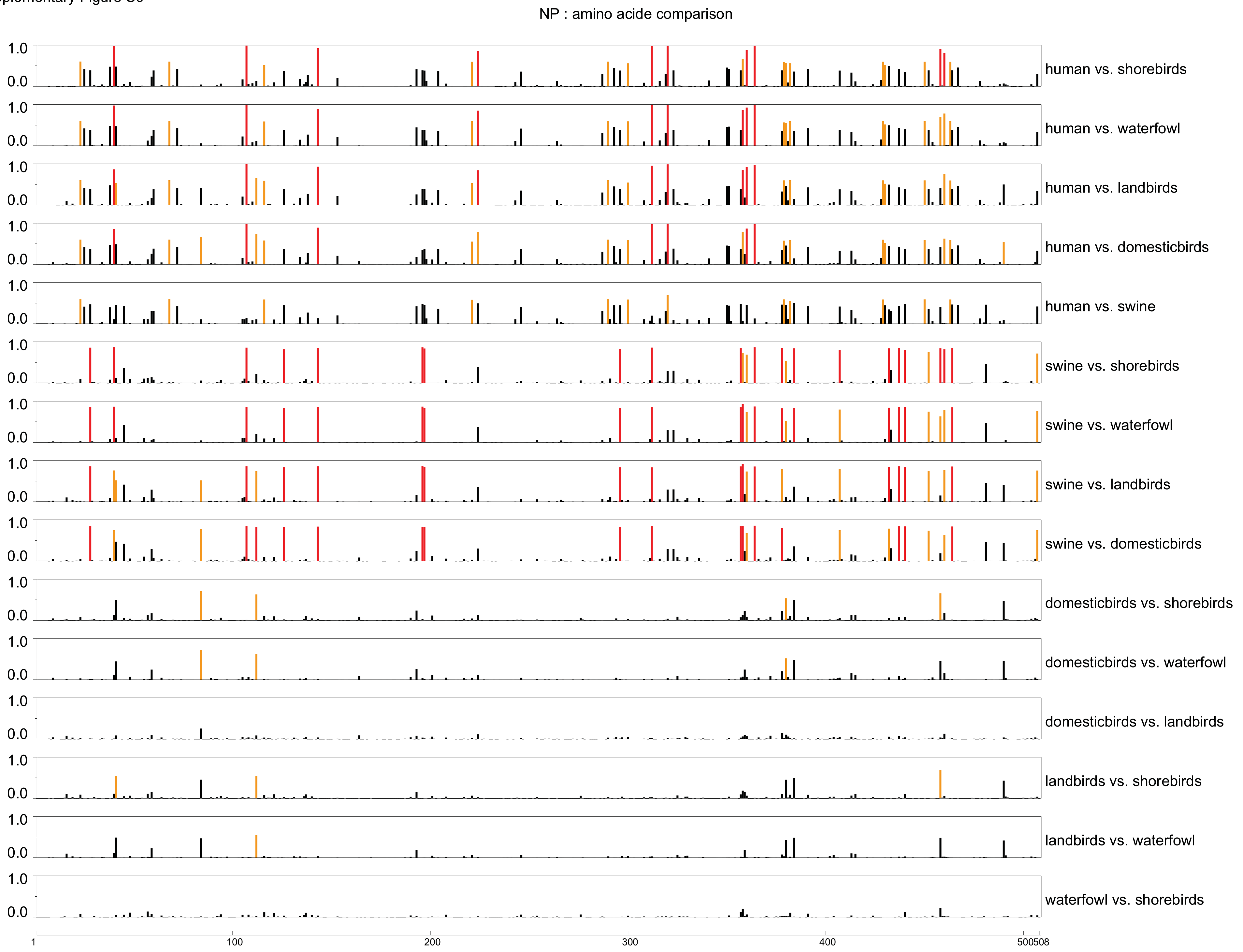

Supplementary Figure S10

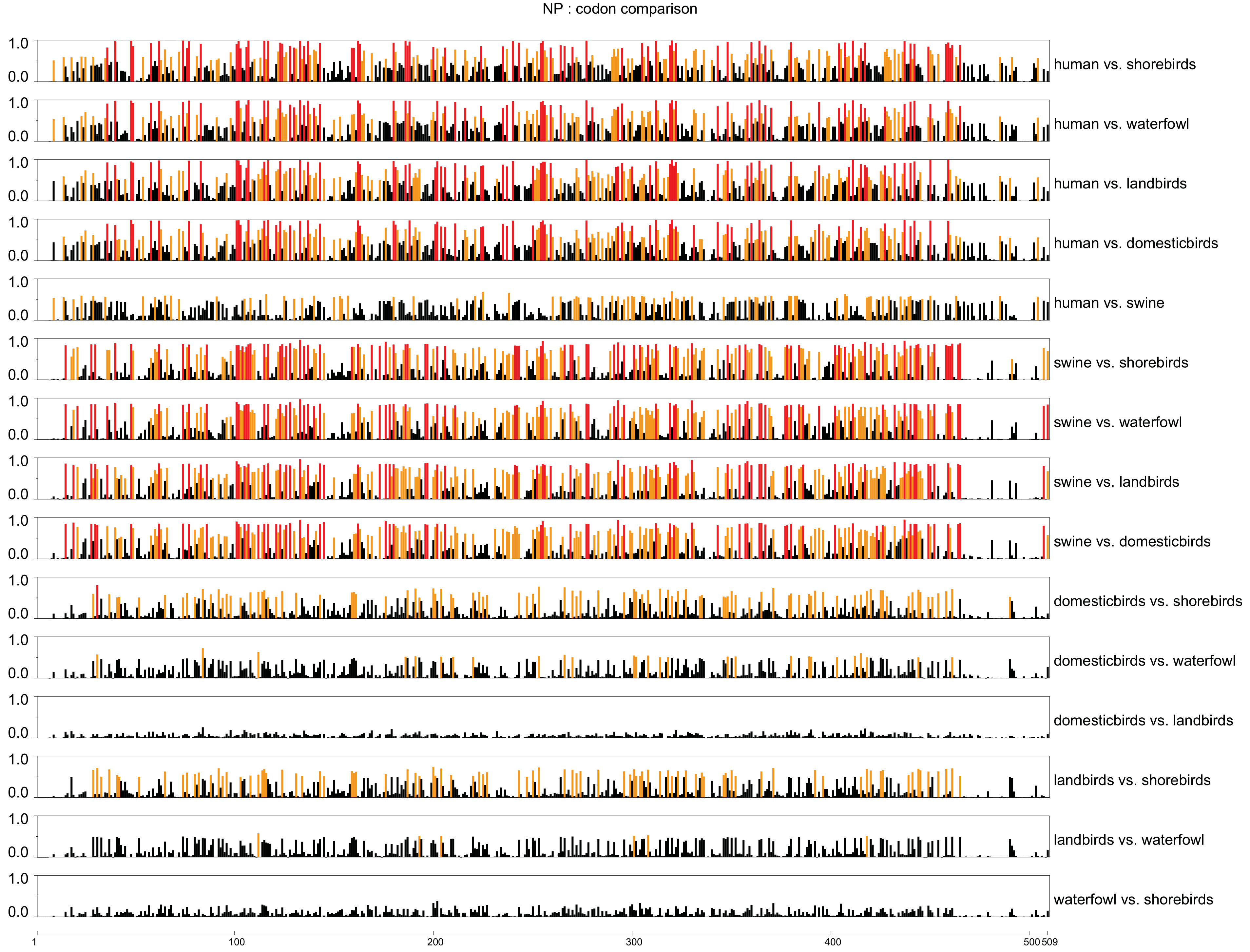

Supplementary Figure S11

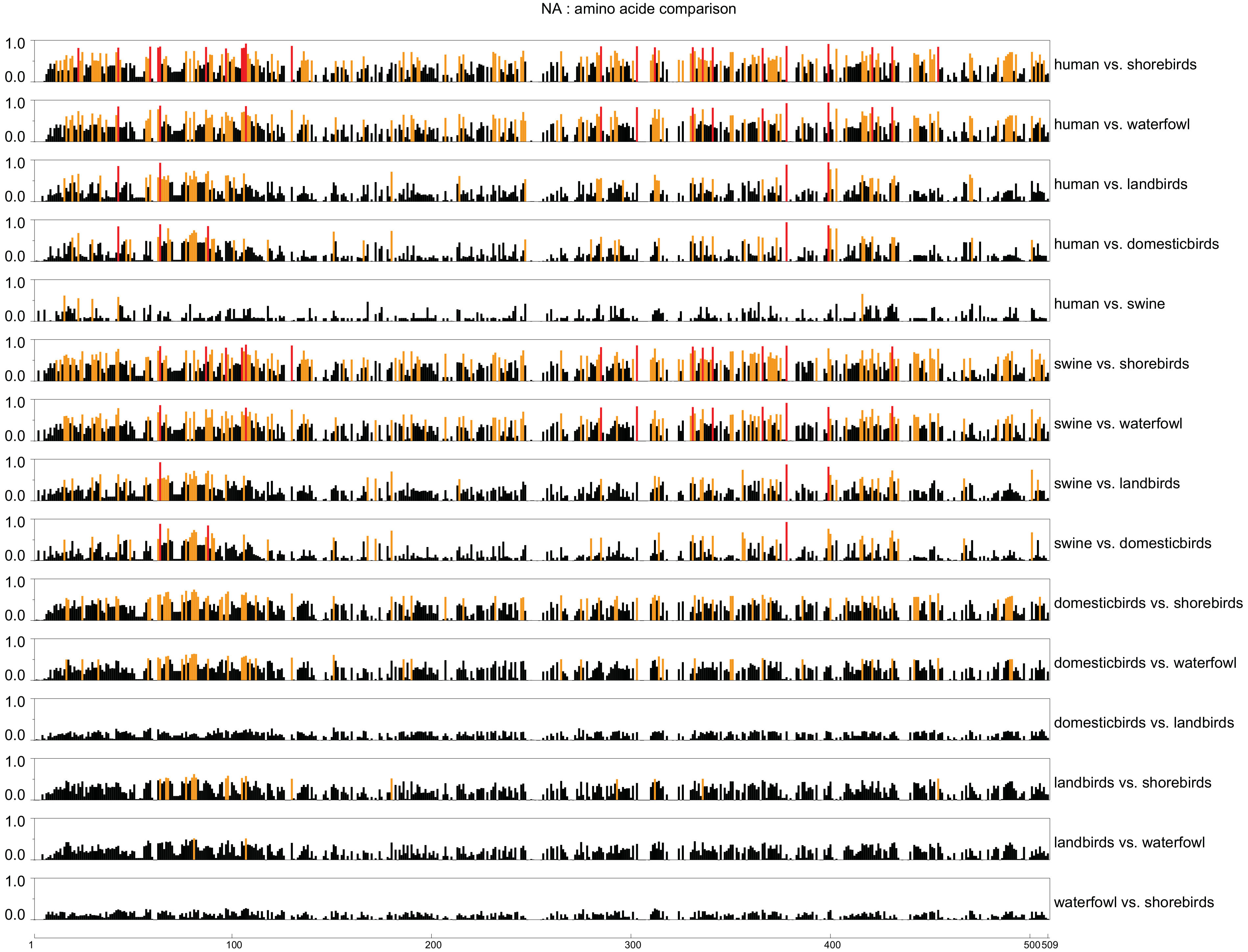

Supplementary Figure S12

NA : codon comparison

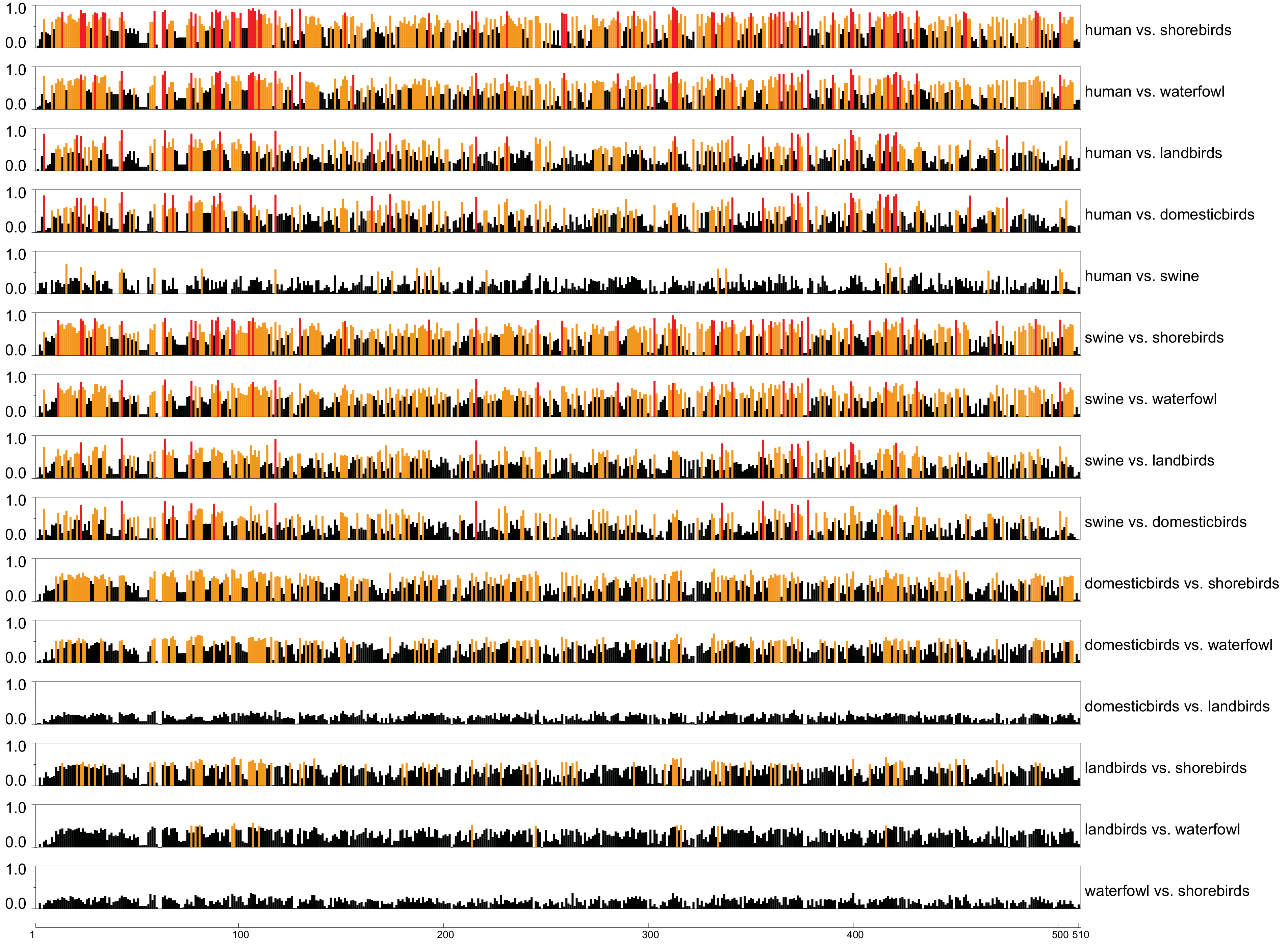

Supplementary Figure S13

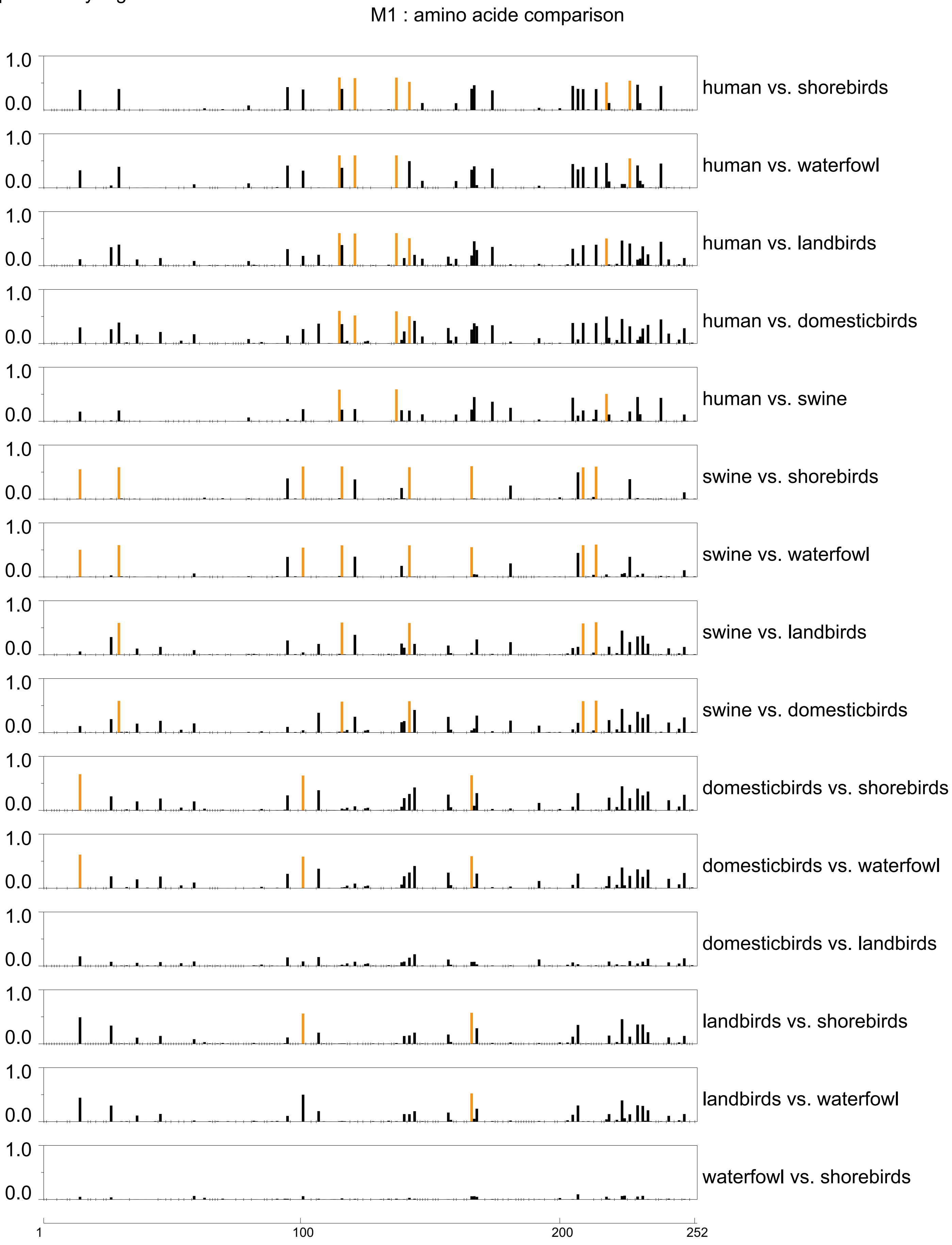

Supplementary Figure S14

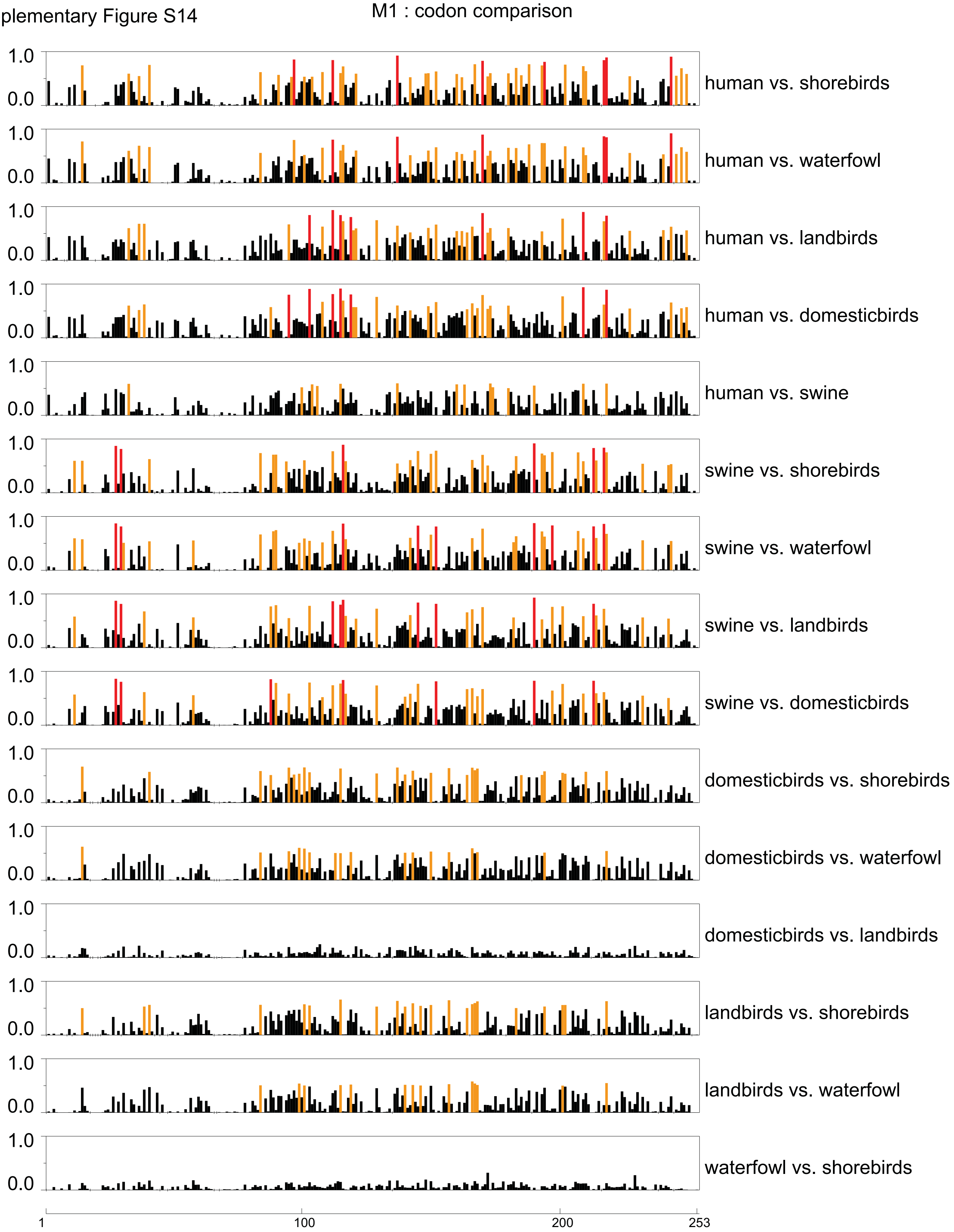

Supplementary Figure S15

NS1 : amino acide comparison

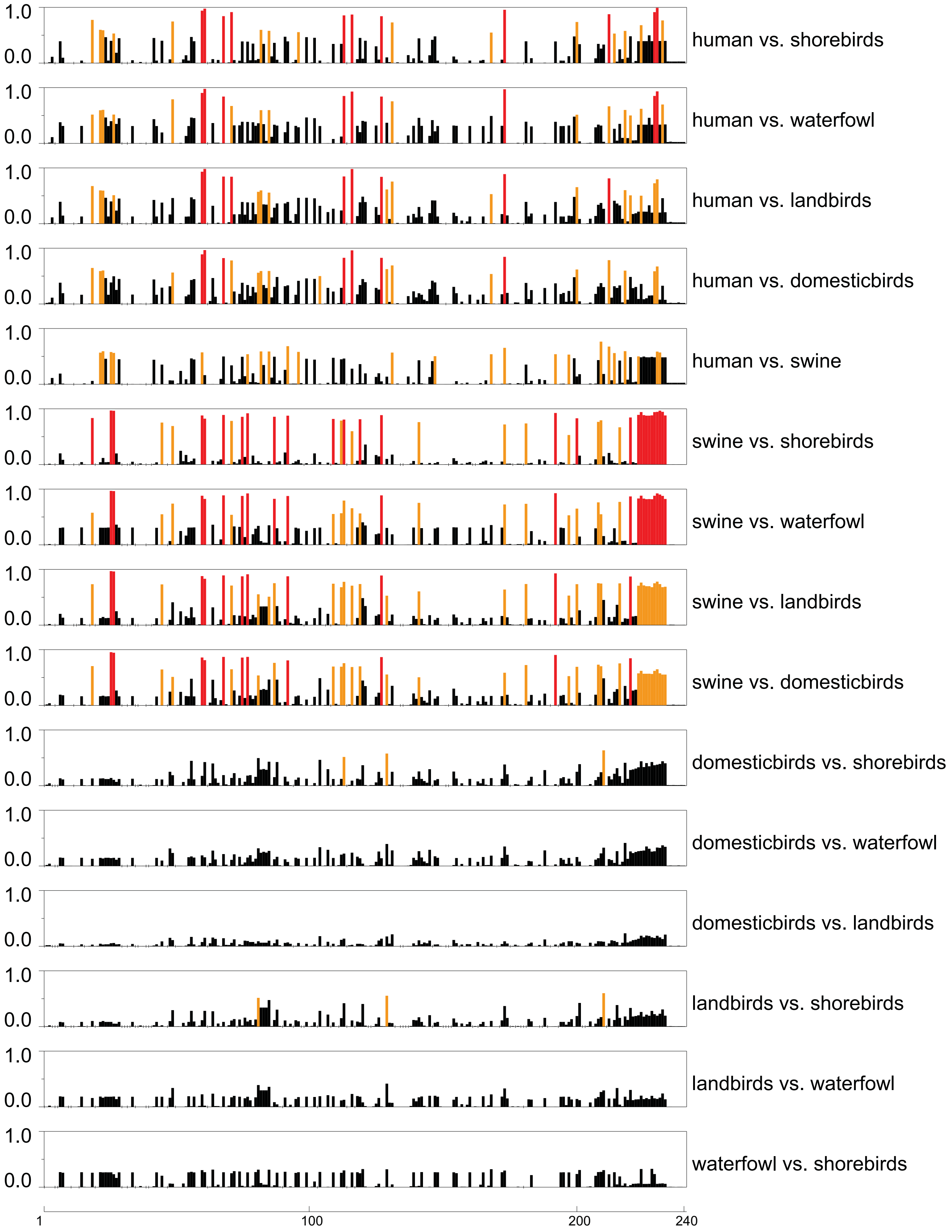

Supplementary Figure S16

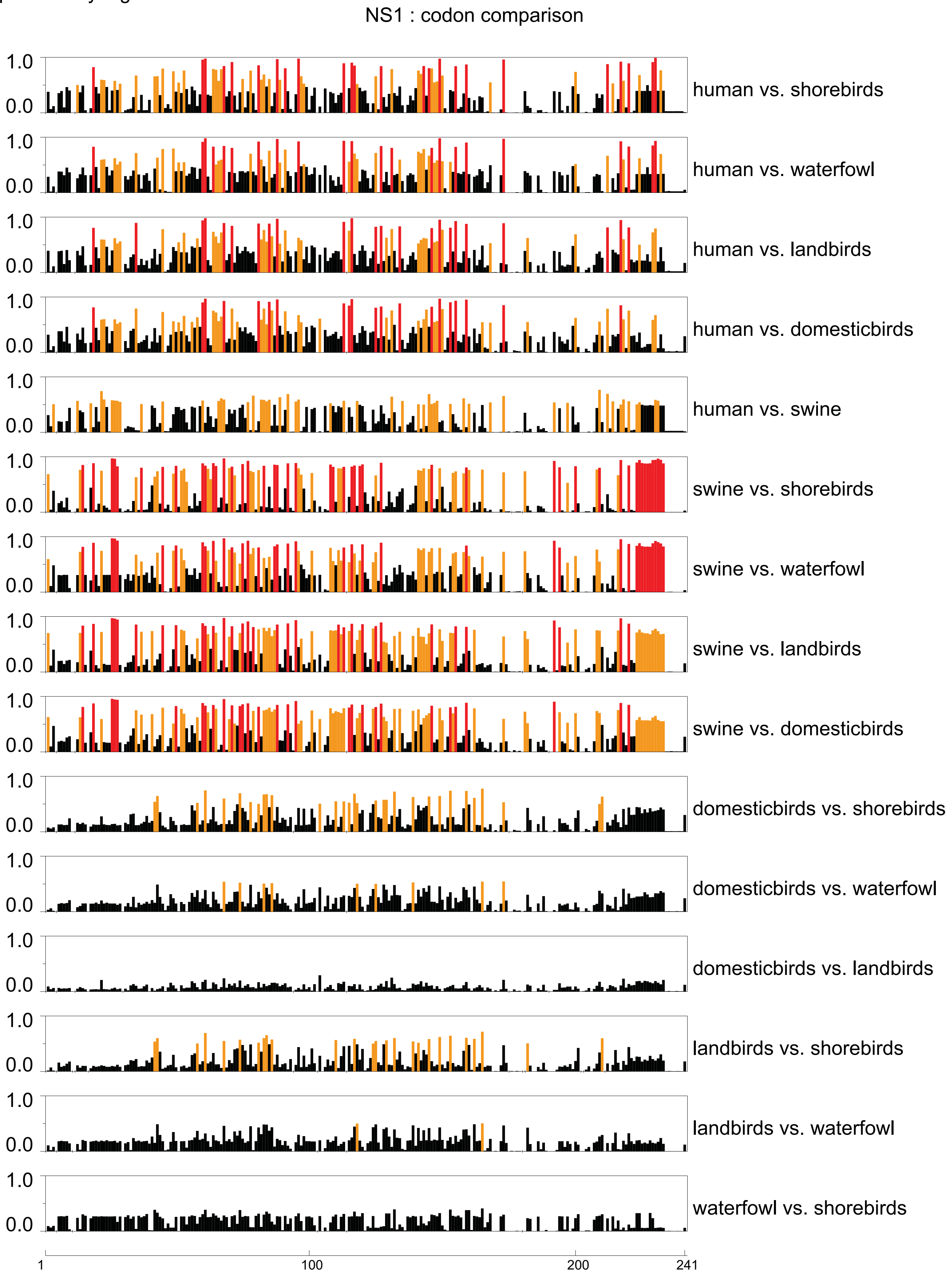

Supplement: Supplementary Information [file srep36839-s1.pdf]
